# Biomarker combinations in predicting sepsis in hospitalized children with fever

**DOI:** 10.1186/s12887-022-03285-3

**Published:** 2022-05-12

**Authors:** Linda Rautiainen, Anna Cirko, Jana Pavare, Ilze Grope, Gita Gersone, Peteris Tretjakovs, Dace Gardovska

**Affiliations:** 1 Department of Pediatrics, Riga Stradins University, Riga, LV1007 Latvia; 2 Childrens Clinical University Hospital, Riga, LV1004 Latvia; 3 Department of Human Physiology and Biochemistry, Riga Stradins University, Riga, LV1007 Latvia

**Keywords:** Fever, Sepsis, Pediatric, Biomarker, Cytokine

## Abstract

Sepsis is among the leading causes of critical illness worldwide. It includes physiologic, pathologic, and biochemical abnormalities, induced by infection. Novel methods for recognizing a dysregulated inflammatory response and predicting associated mortality must be developed. Our aim was to investigate biomarkers that characterize a pro-inflammatory and anti-inflammatory response in patients with fever by comparing predictive validity for sepsis. 165 patients with fever were enrolled in this study, 55 of them had sepsis according to pSOFA criteria. All patients had blood samples drawn at the time of inclusion and after 24 h. CRP, PCT and also IL-6, IL-8 and sFAS levels were significantly higher in patients with sepsis. The AUC of CRP to predict sepsis was 0.799, all the other biomarkers had AUC’s lower than that. Cytokines, when used as a single marker, did not show a significant diagnostic performance We analyzed various models of biomarker combinations. CRP combined with sFAS showed increase in sensitivity in predicting sepsis (88% vs. 83%). The highest AUC was achieved, when CRP, IL-6, sFAS and sVCAM-1 markers were combined 0.830 (95% CI 0.762–0.884) with a sensitivity of 70% and specificity of 84%. vs. 0.799 for CRP alone.

## Background

Sepsis is a “life-threatening organ dysfunction caused by a dysregulated host response to infection,” and it is still among the leading causes of critical illness worldwide [1]⁠. It includes physiologic, pathologic, and biochemical abnormalities, induced by infection, but the the host response to an infecting pathogen is as important, as it can be amplified and dysregulated [1]. The original sepsis definition with at least 2 of systemic inflammatory response syndrome (SIRS) criteria focused on excess of inflammatory reaction [2]⁠. However, now it is recognized to involve pro- and anti-inflammatory response activation is sepsis pathways [3]⁠.

Although, sepsis has been widely studied in recent years, there is still no consensus on how sepsis is clinically defined. International Pediatric Sepsis Consensus Conference defined sepsis as systemic inflammatory response syndrome (SIRS) in the presence or as a result of suspected or proven infection [4]⁠. The definitions for adult sepsis have been updated, but definition of pediatric sepsis remains unchanged. However, there have been research on pediatric sepsis and several new approaches have been proposed, e.g., pediatric Sequential Organ Failure Assessment score (pSOFA) [5]⁠.

As sepsis diagnosis remains challenging and there are few, if any reliable tools in predicting outcome of pediatric sepsis, novel methods for recognizing a dysregulated inflammatory response and predicting mortality must be researched and developed [6]⁠. The SOFA score integrates clinical signs and laboratory values (e.g.,Glasgow Coma Scale, urine output, mean arterial pressure, PaO2/FiO2 ratio, platelet count, serum bilirubin,,, serum creatinine). In addition to SOFA criteria, the new consensus definition of septic shock incorporates a biomarker, serum lactate, [1]⁠. Biomarkers can be measured objectively and serve as measurable indicators of normal biological and pathological processes, or response to a therapeutic intervention [7]⁠. Currently used methods that may aid in the distinction between bacterial and viral infections are primarily white blood cell (WBC) counts, C-reactive protein (CRP) and procalcitonin (PCT) levels. WBC provide some diagnostic value for ruling in serious infection with positive likelihood ratios from 0.87 to 2.43 [8]. ⁠CRP has shown to have fair sensitivity of 75%, specificity of 67% and area under the curve (AUC) of 0.77 for distinguishing patients with sepsis from non-infectious SIRS [9]. PCT has been found to have sensitivity of 77% and specificity of 0.79 for diagnosis of sepsis in critically ill patients Search for biomarkers that could provide a reliable and early estimate of the likelihood of bacterial infection, risk stratification and mortality risk estimate still continues, and currently there is no single biomarker that can accurately predict sepsis [6, 8, 9]⁠.

Our previous study investigated difference patterns of chemokines and cytokines between children with and without serious bacterial infections [10]⁠. As continuation to our previous research, aim of this study was to investigate the same biomarkers that characterize a pro-inflammatory and anti-inflammatory response in patients with fever by comparing predictive validity for sepsis. Biomarker selection was made, based on already published data about their importance in sepsis and systemic inflammatory response, as well as adding biomarkers that have not yet been widely studied in pediatric sepsis. We investigated twelve biomarkers, in children with fever, admitted to hospital from the emergency department at a tertiary level children’s hospital.

## Methods

### Study population

The study was designed as a prospective cohort study, and it combines clinical and laboratory data. Patient recruitment took place from January 2014 to December 2017 in Children’s Clinical University hospital, Riga, Latvia. As an only tertiary children’s hospital of the country, it has around 64,000 pediatric emergency department visits annually. Consecutive patients during this period that met the inclusion criteria, and whose parents or legal guardians consented to the study were recruited.

The inclusion criteria for the study were suspected diagnosis of community acquired infection and patients, that were older than 30 days but younger than 18 years.. Exclusion criteria were the same as in our previous study: antibacterial therapy within the last 48 h, conditions and diseases which are known to be associated with significant changes of inflammatory biomarkers: immunodeficiency, chronic liver or kidney illness, vaccination within 5 days before the start of the illness, congenital metabolic defects, chromosomal anomalies, and use of corticosteroids or immunosuppressant medications. Also, patients with obesity, diabetes mellitus, chronic inflammatory diseases, such as rheumatoid arthritis, systemic lupus erythematosus, vasculitis, inflammatory bowel disease, heart diseases, renal or liver diseases, or malignancies and other diseases which are known to be associated with significant changes of anti- and pro-inflammatory biomarkers, including surgery or trauma within the preceding 30 days, were excluded [10]⁠.

### Clinical protocol and definition

Infection at emergency department and during revision of clinicians was defined based on available clinical, imaging, and on microbiological data, where applicable [11]. Sepsis at the time of inclusion was defined accordingly to pSOFA score⁠. pSOFA uses clinical variables of respiratory (PaO2:FiO2 or SpO2:FiO2), coagulation (platelet count), hepatic (bilirubin levels), cardiovascular (mean arterial pressure by age group or vasoactive drug infusion), neurologic (Glasgow Coma Score) and renal function (creatinine by age group). Each of these variables is scored from 0 to 4, resulting in total score 0–24, and higher scores indicate a worse outcome [5]. In the original publications patients with sepsis were defined as those with confirmed or suspected infection who had an acute rise in the pSOFA score of 2 points or more from up to 48 h before the infection. As patients were recruited from the emergency department and none of the patients had previous organ dysfunction, the pre-infection pSOFA score was assumed to be zero.

### Data collection and laboratory assays

The clinical assessment and physical data were gathered at the emergency department by physicians and nurses, where applicable. For data collection and laboratory assays we used an already established protocol from our previous study [10]. All patients had blood samples drawn at the time of inclusion. According to hospital’s standards full blood count and CRP was measured, as well as other necessary laboratory tests according to the clinical judgement of a physician. For this study blood samples were drawn at the same time with other clinical blood samples for serum centrifugation. Serum was then frozen, and all patients’ samples were analyzed at once. We measured levels of the cytokines and chemokines as in our previous study: interleukin 6 (IL-6), interleukin 8 (IL-8), interleukin (IL-10), interferon gamma (IFN-γ), interleukin-1 receptor antagonist (IL-1ra), soluble apoptosis- stimulating fragment (sFAS), soluble vascular cell adhesion molecule (sVCAM-1), total plasminogen activator inhibitor type 1 (tPAI1), Eotaxin-1, granulocyte colony-stimulating factor (G-CSF), interferon-inducible protein-10 (IP-10), monocyte chemoattractant protein-1 (MCP-1) using Luminex® xMAP® technology, a multiplex assay approach (Luminex 200™, Merck Millipore) [10]. All tests were performed in accordance with the manufacturer’s instructions (Cat#: HSP1MAG-63 K and HTH17MAG-14 K; Milliplex™).

### Ethical considerations

Patients’ parents or legal guardians signed an informed consent to participate in this study. The study protocol has been previously approved by the Committee of Ethics of Riga Stradin’s University (No 2./06.10.2011). Patients’ standard of care was ensured according to hospital guidelines.

### Statistical analyses

Statistical analysis was performed using IBM SPSS 22. For the categorical and continuous variables descriptive statistical methods were used. Categorical variables are shown as numbers and percentages. The Kolmogorov–Smirnov test was used to determine whether the continuous variables followed normal distribution. Data is shown as mean with standard deviation (SD), in case of normal distribution, and it is presented as the medians and interquartile ranges (IQR), if the data did not follow a normal distribution, To test for differences between the compared groups, we used the Mann–Whitney test, and for comparison of related samples we used Wilcoxon signed rank test. Unadjusted and adjusted logistic regression models were calculated to find the best predictors of sepsis. Then, receiver-operating characteristic (ROC) curves were created for each biomarker presenting the area under the curve, including the 95% confidence interval (CI). The Youden’s index was used to determine the best cut-off values for each biomarker to maximize sensitivity and specificity (maximum = sensitivity + specificity −1). A two-tailed *p* value <0.05 was considered statistically significant.

## Results

In total, 165 patients, older than 1 month but younger than 18 years, were enrolled in this study. The baseline characteristics of study population are presented in Table 1.

**Table 1:** Characteristics of study sample

Characteristics of the Study Sample	Sepsis Group (*n* = 55)	Non-sepsis Group (*n* = 110)
**Clinical characteristics**		
Age, months, median (IQR)	78 (33.50–163.00)	31.50 (26.24–40.00)
Male, % (n)	52.7% (29)	42.7% (47)
Day of illness at the time inclusion^a^, median (IQR)	3 (2–3)	3 (3–4)
pSOFA score, % (n)	2 = 38.2% (21) 3 = 27.3% (15) 4 = 14.5% (8) 5 = 10.9% (6) 6 = 1.8% (1) 7 = 1.8% (1) 9 = 3.6% (2) 10 = 1.8% (1)	0 = 86 1 = 24
ICU admission, % (n)	44.4% (24)	1.8% (2)
Length of ICU stay, median (IQR)	3.5 (1–8)	1.5 (1–2)
Required invasive ventilation, % (n)	9.2% (5)	–
Length of hospital stay, days, median (IQR)	13 (9–15)	5 (5–6)
**Laboratory characteristics**		
WBC, median (IQR) x 10^9^/L	10.92 (7.52–19.54)	13.6 (8.07–17.92)
PLT, median (IQR) x 10^12^/L	219.00 (127.25–326.50)	279.00 (236.50–366.75)
CRP, median (IQR), mg/L	162.00 (82.65–262.75)	42.90 (13.21–102.15)
PCT, median (IQR), ng/mL	3.95 (0.69–12.94)	0.34 (0.11–2.28)
Positive blood culture, % (n)	1.8% (1)	–
**Inflammatory cytokine levels**		
Eotaxin, median (IQR), pg/mL	61.77 (46.18–112.62)	83.66 (57.69–121.40)
G-CSF, median (IQR), pg/mL	219.11 (58.56–524.93)	212.88 (94.66–710.43)
IFN gamma, median (IQR), pg/mL	10.72 (4.34–25.08)	15.06 (7.70–53.43)
IL-10, median (IQR), pg/mL	23.03 (11.46–73.61)	37.17 (20.16–81.79)
IL-1ra, median (IQR), pg/mL	38.70 (17.27–143.67)	42.883 (14.20–102.13)
IL-6, median (IQR), pg/mL	48.95 (24.45–235.00)	22.55 (8.63–58.55)
IL-8, median (IQR), pg/mL	16.54 (8.82–36.40)	14.14 (6.91–21.17)
IP-10, median (IQR), pg/mL	786.33 (347.72–2202.61)	975.72 (552.25–2103.36)
MCP-1, median (IQR), pg/mL	393.44 (274.28–703.09)	472.96 (302.29–841.54)
PAI-1, median (IQR), pg/mL	101,564.90 (117.88–150,633.47)	83,516.50 (143.80–163,213.34)
sFAS, median (IQR), pg/mL	3171.12 (2592.33–4280.32)	2738.20 (2154.14–3552.89)
sVACM-1, median (IQR), pg/mL	505,020.07 (1262.56–806,747.45)	352,718.78 (1081.54–1,034,808.95)

^a^Day of illness – day after symptom onset as reported at the time of hospital admission by patients’ caregivers

CRP and PCT levels were significantly higher in patients with sepsis, whereas WBC levels did not have significant difference between sepsis and non-sepsis group. When comparing levels of inflammatory cytokines between sepsis and non-sepsis group, statistically significant differences were seen in IL-6 (*p* < 0.0001), IL-8 (*p* = 0.033) and sFAS (*p* = 0.013)

The area under the curve (AUC) of CRP was 0.799, and at cut-off level of 69.9 mg/L in prediction of sepsis its sensitivity was 83% and specificity 65%. PCT and IL-6 had lower AUC’s than CRP in predicting sepsis but their sensitivity was higher – 87% for PCT and 86% for IL-6.

**Fig. 1:**
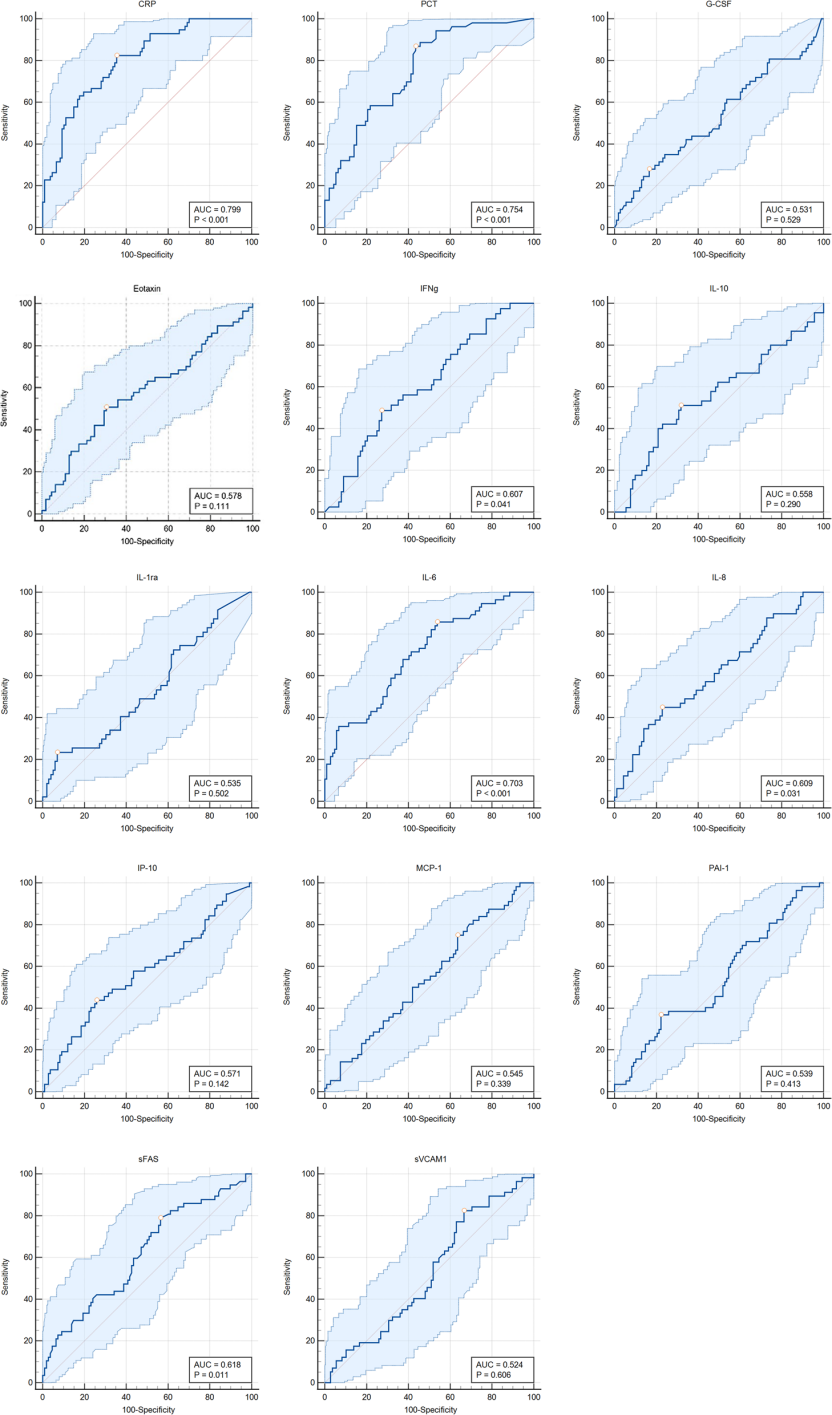
ROC curves with 95% CI, and Youden’s index, AUC with p values for each single biomarker

ROC curves with 95% CI, AUC and p values as well as the point of Youdens index for each biomarker is depicted in Fig. 1. Cutoff levels, together with sensitivity and specificity are shown in Table 2. All other cytokines, when used as a single marker, did not show a significant diagnostic performance.

**Table 2:** Cutoff values for CRP, PCT and other biomarkers in predicting sepsis, including their sensitivity and specificity, positive predictive value (PPV), negative predictive value (NPV) with 95% confidence intervals

Variable	Cutoff Value	Sensitivity, % (95% CI)	Specificity, % (95% CI)	PPV % (95% CI)	NPV % (95% CI)
CRP, mg/L	69.9	83 (70–91)	65 (55–74)	70 (64–76)	79 (68–87)
PCT, ng/mL	0.43	87 (75–95)	57 (46–67)	67 (62–72)	81 (69–90)
G-CSF, pg/mL	61.58	28 (18–42)	83 (75–90)	62 (48–75)	54 (49–58)
Eotaxin, pg/mL	61.77	51 (37–64)	69 (60–78)	62 (53–71)	59 (51–66)
IFN gamma, pg/mL	8.79	49 (33–65)	73 (62–82)	65 (55–73)	59 (52–66)
IL-10, pg/mL	23.03	51 (36–66)	68 (58–78)	62 (52–70)	58 (51–65)
IL-1ra, pg/mL	213.75	23 (12–38)	93 (86–97)	77 (59–88)	55 (51–59)
IL-6, pg/mL	18.30	86 (74–94)	46 (36–56)	61 (57–66)	77 (62–87)
IL-8, pg/mL	21.90	45 (31–60)	77 (67–85)	66 (56–75)	58 (52–65)
IP-10, pg/mL	569.57	44 (31–58)	74 (65–82)	63 (52–72)	57 (51–63)
MCP-1, pg/mL	673.92	75 (62–86)	37 (27–46)	54 (49–50)	60 (47–71)
PAI-1, pg/mL	135	37 (24–51)	78 (69–85)	63 (51–73)	55 (50–61)
sFAS, pg/mL	2538.29	79 (66–89)	44 (34–53)	58 (52–63)	65 (53–75)
sVCAM-1, pg/mL	868,886.52	83 (70–91)	33 (25–43)	55 (51–60)	66 (51–79)

**Fig. 2:**
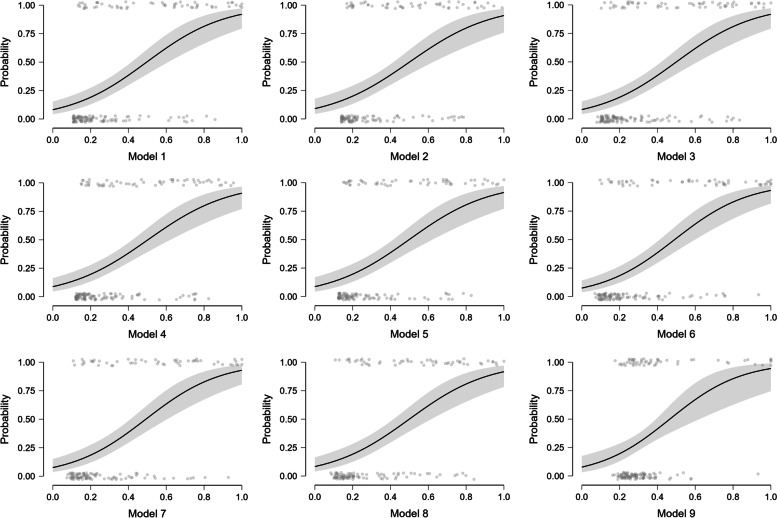
Logistic regression model graphs with 95% CI for different biomarker models

To test for added diagnostic performance, we analyzed efficacy of different models of biomarker combinations in sepsis diagnostics. When sFAS was added to CRP, it increased diagnostic sensitivity to 88% (95% CI 76–95) CRP, combined with IL-6, sFAS and sVCAM-1 had the highest AUC of 0.830 (95% CI 0.762–0.884) with a sensitivity of 70% and specificity of 84%. Results of the model analysis are shown in Table 3 and logistic regression models with 95% CI in Fig. 2.

**Table 3:** Sensitivity, specificity, positive predictive values (PPV) and negative predictive values (NPV) and 95% confidence intervals (CI) of biomarker combinations to predict sepsis

		Sensitivity % (95% CI)	Specificity % (95% CI)	PPV % (95% CI)	NPV % (95% CI)
Model 1	CRP, IL-6	70 (56–81)	83 (74–89)	80 (72–86)	73 (64–80)
Model 2	CRP, sFAS	88 (76–95)	64 (54–73)	71 (65–76)	84 (72–91)
Model 3	CRP, sVCAM-1	83 (70–91)	65 (55–74)	70 (64–75)	79 (67–87)
Model 4	CRP, PCT, IL-6	73 (59–84)	75 (65–83)	75 (67–81)	74 (64–81)
Model 5	CRP, IL-6, sFAS, sVCAM-1	70 (56–81)	84 (75–90)	81 (73–87)	73 (65–81)
Model 6	CRP, PCT, IL-6, sFAS, sVCAM-1	69 (55–80)	82 (72–89)	79 (71–85)	73 (64–80)
Model 7	CRP, PCT, sVCAM-1	69 (56–82)	79 (70–87)	77 (69–84)	72 (63–80)
Model 8	CRP, PCT, sFAS	67 (54–80)	83 (75–91)	81 (72–87)	73 (64–79)
Model 9	IL-6, sFAS, sVCAM-1	53 (40–67)	80 (72–87)	74 (64–81)	64 (56–70)

**Fig. 3:**
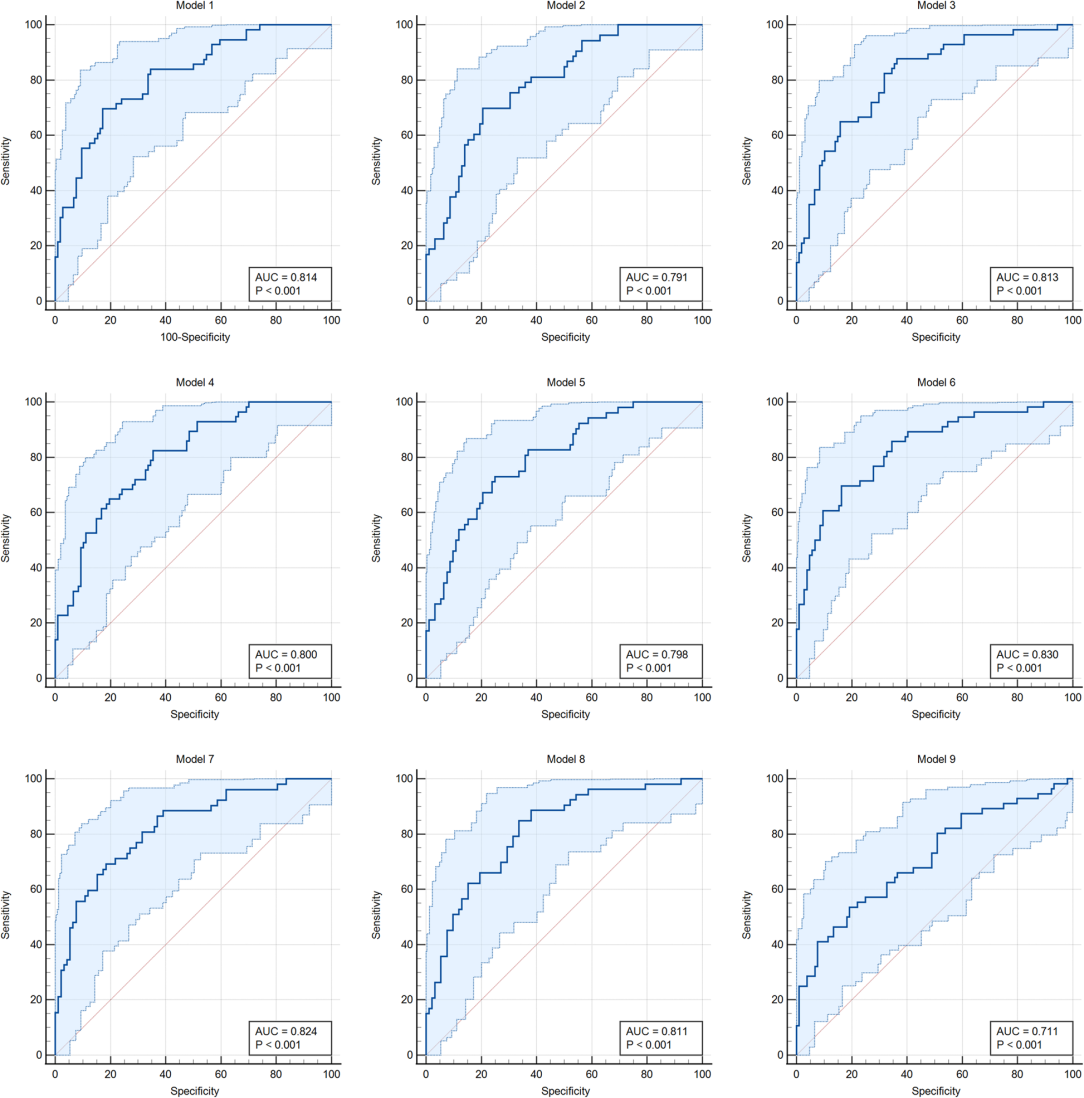
ROC curves (with 95% CI) for models of biomarker combinations in predicting sepsis

The ROC curve analysis with 95% CI and AUC of biomarker combinations are shown in Fig. 3.

## Discussion

As sepsis is heterogenous condition, a dysregulation of pro- and anti-inflammatory responses, its diagnostics remains challenging. It is especially challenging in children, due to low incidence of bacterial infections (prevalence of serious bacterial infections in children, presenting with fever to ED varies from 4.5 to 29.3%, depending on the study), non-specific presentation and risk of rapid deterioration [8, 9]. CRP has been used for many years in sepsis diagnostics, its specificity has been challenged in recent years, as it increases also in cases of trauma, ischemia, burns and other inflammatory conditions, but elevated concentrations of CRP are correlated with increased organ failure risk and mortality [12]⁠. Depending on whether clinicians want to rule out or confirm sepsis, different cut-off values should be used [9]⁠. In our patient group, patients with sepsis had significantly higher levels of CRP that patients without sepsis; at a cut-off level of 69.9 mg/L it had a sensitivity of 82% and specificity of 64% in predicting sepsis. PCT; together with CRP, is also widely used, it has been proposed as more specific and better prognostic marker as CRP ⁠. A meta-analysis has showed a sensitivity of 76% and specificity of 69% in detecting sepsis [13]. Our data showed 87% sensitivity and 57% specificity at a cut-off level of 0.43 ng/mL. Since Sepsis-3 definition has been adopted in 2016, many studies have re-evaluated the performance of PCT in diagnosing sepsis. When sepsis is defined by Sepsis-3 criteria, PCT had a sensitivity of 74.8% and a specificity of 63.8% with respect to diagnosing sepsis in emergency patients. We used pSOFA criteria, which is adapted from Sepsis-3 SOFA score for pediatric patients, and it could explain why the specificity of PCT in diagnosing sepsis was even lower than CRP’s.

IL-6 has been widely studied in both, adult and pediatric populations [14]⁠. Franco et al. report in their analysis, that in adult population IL-6 had a 66% sensitivity in sepsis prediction [15]⁠. A meta-analysis by Shahkar et al. showed IL-6 sensitivity of 79% and specificity of 82% in predicting neonatal sepsis [16]⁠. Colleagues Pavare et al. have reported similar results, in children with SIRS – IL-6 had an AUC of 0.869 in predicting bacteremia, and the sensitivity using a cut-off level of 58.7 pg/ml was 80%. In our study population the sensitivity was 86% and specificity 46%at a cut-off level of 18.3 pg/ml; this could be explained by different clinical criteria (pSOFA instead of SIRS) used in our study. Also, differences in neonatal, adult and children’s populations could be due to different rates of bacterial infections – children with fever have lower prevalence rate of bacterial infection when compared to adults and neonates with suspected infection.

sFAS has been studied as a biomarker for identification of patients with sepsis, especially in a combination with other markers, it has showed promising results to be considered as a complementary marker for the diagnosis of sepsis [17]⁠. Punyadeera et al. in their biomarker panel analysis showed that levels of sFAS were higher in patients with severe sepsis and septic shock, also it positively correlated with SOFA score [17]⁠. Hahn et al. have published a study about biomarkers’ association with clinical outcome; in this study sFAS was associated with mortality in septic patients, and the median concentration in that population was 12,168 pg/ml [18]⁠. In our study the median level of sFAS in septic patients was 3171.12 pg/ml, the difference could be explained with a different study population (our patients were children and there was no mortality in our study population). sFAS has not been widely studied in pediatric patients with sepsis, and it is not yet used in clinical practice. sVCAM-1 as an endothelial dysfunction marker could reflect outcome of sepsis. In a study, published by Yingying Fang, sVCAM levels were significantly increased in sepsis non-survivors. This study populations had sVCAM-1 levels of 339.7 mcg/ml in patients with severe sepsis and 421.2 mcg/ml in patients with septic shock [19]⁠. When converted to the same units, our patients with sepsis had sVCAM-1 levels of 505.02 mcg/ml. However, these values are not directly comparable due to different study methodology and differences in laboratory assays used. Also, Shapiro et al. have found association between levels of sVCAM-1 and sepsis severity as well as its correlation with SOFA score at the time of admission [20]⁠. There have been no large studies about utility of sVCAM-1 as a diagnostic and prognostic marker in pediatric population.

CRP and PCT have been largely studied as diagnostic markers in sepsis in both, children and adult populations, IL-6 have been studied in children and neonates. Other cytokines and chemokines have been largely studied in pediatric sepsis as markers of systemic inflammation, prognostic markers for organ failure and immunosuppression, not focusing on differentiating patients with sepsis, so data comparison remains challenging [21, 22]. Also, it has been noted that some cytokines perform better as diagnostic markers in pediatric population than in adults, e.g., IL-27 and IL-8 [23]. As our study population had great age variations (from 1 months up to 18 years of age), we can speculate that results could be different if analyzed in more detailed age groups. Research on possible novel biomarker combinations to improve sepsis diagnostics is ongoing. Lin Ruan has published a meta-analysis and a systematic review about biomarker combination in predicting neonatal sepsis, in this study PCT together with CRP improved accuracy of diagnosis of neonatal sepsis [24]⁠. All models of cytokine and chemokine combinations with CRP and PCT had AUC’s above 0.711, however, only CRP and sFAS combination had a sensitivity of 88%, and CRP and sVCAM-1 combination – 83%. Biomarker combinations together with various clinical score systems perform better in sepsis diagnostics than a single biomarker, however, the cost-effectiveness of these combinations is not known and have not been widely studied [9, 25]⁠. If a combination of CRP and sFAS increases sensitivity of sepsis diagnosis to 88% compared to 87% for PCT alone as in our study population, routine use of these biomarker combinations should be carefully evaluated also from economical aspects.

Positive blood culture is not required to meet sepsis criteria, but it remains the gold standard for diagnosing sepsis. In this study population only 1.8% of sepsis patients had positive blood culture. The most important factor for optimal blood culture collection is adequate volume of blood for culture. It is recommended to draw 20–30 mL blood from adult patients from 2 different venipuncture sites [26]⁠. It is challenging to obtain adequate volumes of blood for culturing, as children have lower blood volume compared with adults, as well as technical difficulties in drawing 2 cultures from different venipuncture sites. As much as 60% children have low-level bacteremia, <1 cfu/mL, which can be easily missed, if a small amount of blood is sent for culture [27]⁠. The low number of positive blood cultures could be explained by small volumes of blood drawn for cultures as at the time of the study there was no standardized protocol for volume of blood required for culturing in pediatric patients of different ages. We enrolled patients that had not received antibacterial therapy in the previous 48 h, but we did not gather information about antibacterial therapy before this timeframe, we do not know, if possible antibacterial therapies more than 48 h before enrollment could have impact on blood culture results.

Our study had several limitations. This was a single center study, without a case-control match. Also, different sepsis definitions could have impact on biomarker studies. We had sepsis defined by pSOFA score, and it is possible, that different sepsis definitions would have showed different results. As the study population is small, data extrapolation to larger populations is limited. Some of the cytokines and chemokines have not been largely studied in pediatric sepsis, so comparison and interpretation of our results is difficult.

We found that biomarker combinations could be valuable in diagnosing sepsis in children with fever. Based on these results, sFAS and sVCAM-1 could be proposed for further research as additional biomarkers in sepsis diagnostics in children under 17 years of age. As well as diagnostic cut-off values, it would be important to investigate cut-off values of these biomarkers and their combinations for ruling out sepsis in febrile children.

## Conclusions

CRP, combined with sFAS showed increased sensitivity in predicting sepsis than CRP alone, and CRP, PCT, IL-6, sFAS and sVCAM-1 combined had the highest AUC compared to other biomarker combination models.

## Data Availability

All data generated or analysed during this study are included in this published article.

## Abbreviations

SIRS: Systemic inflammatory response syndrome
SOFA: Sequential Organ Failure Assessment score
WBC: White blood cells
CRP: C-reactive protein
PCT: Procalcitonin
IL-6: Interleukin 6
IL-8: Interleukin 8
TNF-α: Tumour necrosis factor alpha
IL-10: Interleukin 10
IL-4: Interleukin 4
IFN-γ: Interferon gamma
CD64: Cluster of differentiation 64
sICAM: Soluble intercellular adhesion molecules
IQR: Interquartile range
sFAS: Soluble apoptosis- stimulating fragment
sVCAM-1: Soluble vascular cell adhesion molecule
tPAI1: Total plasminogen activator inhibitor type 1
G-CSF: Granulocyte colony-stimulating factor
IL-1ra: Interleukin-1 receptor antagonist
IP-10: Interferon-inducible protein-10
MCP-1: Monocyte chemoattractant protein-1
SD: Standard deviation
IQR: Interquartile range
ROC: Receiver-operating characteristic
CI: Confidence interval
ICU: Intensive care unit
AUC: Area under the curve
PPV: Positive predictive value
NPV: Negative predictive value
